# The regulatory role of lncRNA in tumor drug resistance: refracting light through a narrow aperture

**DOI:** 10.32604/or.2024.053882

**Published:** 2025-03-19

**Authors:** HENG ZHANG, XIAO YANG, YUJIN GUO, HAIBO ZHAO, PEI JIANG, QING-QING YU

**Affiliations:** 1 Department of Laboratory, Shandong Daizhuang Hospital, Jining, 272051, China; 2 Department of Anesthesiology, Affiliated Hospital of Jining Medical University, Jining, 272000, China; 3 Department of Clinical Pharmacy, Jining No.1 People’s Hospital, Jining, 272002, China; 4 Department of Oncology, Jining No.1 People’s Hospital, Jining, 272002, China; 5 Translational Pharmaceutical Laboratory, Jining No.1 People’s Hospital, Jining, 272002, China

**Keywords:** Long chain non coding RNA (LncRNA), Gene regulatory mechanisms, Tumor resistance, Chemotherapy

## Abstract

As living conditions improve and diagnostic capabilities advance, the incidence of tumors has increased, with cancer becoming a leading cause of death worldwide. Surgery, chemotherapy, and radiotherapy are the most common treatments. Despite advances in treatment options, chemotherapy remains a routine first-line treatment for most tumors. Due to the continuous and extensive use of chemotherapy drugs, tumor resistance often develops, becoming a significant cause of treatment failure and poor prognosis. Recent research has increasingly focused on how long stranded non-coding RNAs (LncRNAs) influence the development of malignant tumors and drug resistance by regulating gene expression and other biological mechanisms during cell growth. Studies have demonstrated that variations in lncRNA expression levels, influenced by both interpatient variability and intratumoral genetic and epigenetic differences, are closely linked to tumor drug resistance. Therefore, this review advocates using lncRNA as a framework to investigate the regulation of genes associated with drug resistance, proposing lncRNA-targeted therapeutic strategies to potentially increase the efficacy of chemotherapy, improve patient outcomes, and guide future research directions.

## Introduction

With changes in living conditions and advancements in detection technology, the incidence rate of tumors is increasing, and tumors are becoming a more prominent cause of death [1]. According to the latest statistics of the global tumor database, the global incidence rate and mortality of tumors are growing rapidly [2]. Early malignant tumors lack characteristic clinical symptoms, and most diagnosed patients have already experienced metastasis, making them ineligible for direct surgical treatment. Therefore, chemotherapy has become an important way to treat tumors. However, drug resistance leads to reduced chemotherapy sensitivity in patients with advanced malignant tumors, significantly reducing treatment effectiveness [3,4]. The development of tumor drug resistance can be categorized into primary resistance and acquired resistance.

Based on the resistant characteristics of tumor cells, acquired resistance can be further classified into two subtypes: primary drug resistance (PDR) and multidrug resistance (MDR) [5]. The drug resistance spectrum describes PDR as being insensitive only to the initial drug exposure but still responsive to other drugs. MDR occurs when tumor cells, initially resistant to one drug, also develop resistance to other drugs with different structures and mechanisms. Under these circumstances, simply replacing or combining chemotherapy drugs often proves suboptimal and increases adverse reactions. Tumor drug resistance is a complex process involving multiple factors, drug elimination [6–9], signaling pathways [10–12], and genes [13].

Approximately 2% of the human genome encodes proteins, while the remaining 98% consists of non-coding RNA (ncRNA). These include microRNAs (19–24 bp), PIWI-interacting RNAs (26–31 bp), and transcriptional regulator RNAs (17–18 bp). Long non-coding RNAs (lncRNAs) exceeding 200 bases in length are longer and more complex in synthesis and structure than smaller ncRNAs. Small ncRNAs are primarily expressed through specific gene binding, whereas lncRNAs are mainly transcribed by RNA polymerase II, with a small portion also produced by RNA polymerase III [14]. The small ncRNA sequence is highly conserved, but lncRNA has significant heterogeneity and spatiotemporal specificity [15–17]. The structural complexity of lncRNAs suggests they perform crucial biological functions akin to the ‘dark matter’ of life [18]. LncRNAs have long been regarded as “transcriptional noise”, but as research has progressed, their regulation of gene expression has received increasing attention. To date, only a few lncRNAs have been identified, and their role in tumorigenesis and development has not been fully elucidated.

The lncRNAs regulate gene expression at multiple levels, including transcriptional, post-transcriptional, and epigenetic, and play an important role in cancer development, invasion, and metastasis. LncRNAs modify the epigenetic landscape by recruiting chromatin complexes to specific gene loci. LncRNA Xist/RepA induces X chromosome inactivation by recruiting PRC2 to the X chromosome [19]. At the transcriptional level, certain lncRNAs, including promoter-associated RNAs (paRNAs) and enhancer RNAs (eRNAs), directly interact with target genes, exerting either positive or negative regulatory effects. paRNAs regulate gene expression in the proximal promoter regions, whereas their deletion significantly reduces the expression of target genes. Post-transcriptional modifications include splicing, editing, translation, substitution, and degradation [20]. The transcription of lncRNA has spatiotemporal specificity, and certain external signals act as “switches” to precisely regulate the transcription of lncRNA. Therefore, the transcription of certain lncRNAs provides important clues for developing and differentiating certain tissues. As crucial molecular signals, lncRNAs serve guiding, constraining, baiting, and scaffolding roles, mediating various physiological events and diseases, including tumor development, through downstream cascading reactions. Proteins provide scaffolding for numerous important signaling molecules, while lncRNAs are closely associated with a wide range of biological processes. The expression of these finely regulated RNA molecules makes signaling pathways and interactions between related molecules specific.

LncRNAs and their target molecules or regulation mechanisms targeting biological functions in tumors are also important in tumor drug resistance, as shown in Table 1. One important lncRNA, LUCAT1, is significantly overexpressed in human colorectal cancer tissues [21]. *In vivo* ubiquitination studies indicate that silencing LUCAT1 increases the protein stability of UBA52, although it does not significantly affect mRNA levels [22]. To a large extent, p53 is also regulated by a ubiquitin ligase, MDM2, which binds p53 to promote its degradation. Inhibition of LUCAT1 binding to UBA52 and activation of the UBA52-MDM2-p53 pathway in colorectal cancer knockout mice had significantly elevated expression in colorectal cancer and inhibited colorectal cancer development [21]. Therefore, the LUCAT1/miR-134-5p complex shows high expression levels in gastric cancer tissues [22]. Accordingly, LUCAT1 regulates the proliferation, migration, and invasion of hepatocellular carcinoma cells and esophageal cancer [23,24]. LUCAT1 functions as a ceRNA in hepatocellular carcinoma tissues, binding to and downregulating miR-181d-5p, thereby enhancing cellular proliferation, migration, and invasion [25]. H19, a cancer-embryo-associated lncRNA located on chromosome 11p15.5, acts as a pro-oncogene in many tumors. In gastric cancer, its abnormal upregulation directly inactivates the p53 protein, thereby promoting the proliferation of gastric cancer cells [26]. H19 inhibits colorectal cancer by upregulating miR-29b-3p expression, thus suppressing tumor growth [27]. Knockdown of the CASC15 gene significantly reduced the proliferation and invasiveness of gastric cancer cells [28]. CASC15 promotes EMT, and CASC15 overexpression is closely related to gastric cancer invasion and metastasis by regulating the CASC15/miR-33a-5p/ZEB1 pathway [28]. It has been shown that both LINC01503 and early growth response protein 1 (Egr-1) are significantly higher in the serum of patients with gastric cancer and the correlation with LINC01503 levels in gastric cancer tissue [29]. Further research found that Egr-1-activated LINC01503 can superficially silence the expression of bispecific phosphate 5/cyclin-dependent kinase inhibitor 1A [29]. It promotes gastric cancer cell cycle and progression. KCNMB2-AS1 is highly expressed in esophageal cancer tissues and directly interacts with miR-3194-3p, potentially by upregulating glycogen phosphorylase L [30,31]. XLOC was also upregulated in esophageal cancer tissues, and further experiments showed that XLOC_001659/miR-490-5p/PIK3CA might be a new molecular target for esophageal cancer [32]. The lncRNA BBOX1-AS1 was highly expressed in esophageal cancer, and the knockdown of miR-361-3p promoted COL1A1 expression, promoting esophageal cancer progression [33]. Silencing BBOX1-AS1 could impair cellular activity and migration capacity, induce cell apoptosis, and suppress cancer cell formation *in vivo* [33].

**Table 1:** LncRNA contributes to cancer initiation and progression by regulating the expression of cancer genes

LncRNA	Cancer types	Expression in cancer	Target molecules	Biological function	References
LUCAT1	Colorectal cancer	Up-regulation	P53	Inhibition of LUCAT1 expression leads to an increase in p53 expression, the effects of cell cycle blockade and apoptosis	[21]
	Gastric cancer	Up-regulation	MiR-134-5p	Upregulation of LUCAT1 or downregulation role of miR-134-5p in gastric cancer	[22]
	Liver cancer	Up-regulation	MiR-181d-5p	Overexpression of LUCAT1 or inhibition of miR-181d-5p enhanced HCC cell proliferation, migration and invasion	[24]
	Esophageal cancer	Up-regulation	DNA methyltransferase 1	Regulating the stability of ubiquitination degradation of DNA methyltransferase 1, inhibiting the expression of tumor suppressor factors	[24]
H19	Gastric cancer	Up-regulation	P53	Upregulation of H19 directly inactivates p53 and promotes proliferation of gastric cancer cells	[26]
	Colorectal cancer	Up-regulation	MiR-29b-3p	Downregulation of H19 upregulates the expression of miR-29b-3p, inhibiting the proliferation and progression of colorectal cancer cells	[27]
CASC15	Gastric cancer	Up-regulation	MiR-33a-5p	Upregulation of CASC15 leads to the expression of miR-33a-5p downregulation, regulating ZEB1’s carcinogenic effect and promoting the development of gastric cancer	[28]
LINC 01503	Gastric cancer	Up-regulation	Bispecific phosphate 5	LINC01503 silences the expression of bispecific phosphate 5 and CDKI 1A, promoting the cell cycle and progression of gastric cancer	[29]
KCNMB2-AS1	Esophageal cancer	Up-regulation	MiR-3194-3p	KCNMB2-AS1 binds to miR-3194-3p to upregulate the expression of glycogen phosphorylase L, promoting the occurrence and development of esophageal cancer	[31]
XLOC_001659	Esophageal cancer	Up-regulation	MiR-490-5p	After the upregulation of XLOC_001659, miR-490-5p is downregulated, targeting the upregulation of PIK3CA and the proliferation and invasion of esophageal cancer	[32]
BBOX1-AS1	Esophageal cancer	Up-regulation	MiR-361-3p	Upregulation of BBOX1-AS1 leads to downregulation of miR-361-3p expression, increasing the expression of COL1A1 and the progression of esophageal cancer	[33]

Resistance of tumor cells to chemotherapeutic drugs is an important cause of tumor treatment failure. The emergence of tumor resistance is a complex issue involving multiple processes such as drug metabolism [6], cell damage repair [7], cell cycle [12], and multiple genes [13]. Regardless of whether secondary resistance is congenital or acquired, abnormal activation of intracellular signaling pathways is closely associated with tumor resistance. The lncRNAs, a recently discovered class of non-coding RNAs, possess significant biological functions, including the regulation of several proto-oncogenes. lncRNAs alter the efflux system, enhance drug metabolism, and affect the cell cycle [21–26], thereby inducing abnormal apoptosis [22,23] and promoting drug resistance through epithelial-mesenchymal transition (EMT) [27,28]. In addition, lncRNAs with targeted binding sites can form competing endogenous RNAs (ceRNA) with microRNAs (miRNAs) to participate in drug resistance [29–31]. LncRNAs and proteins regulate multiple gene expression during and after transcription. This review proposes utilizing LncRNA as a starting point to investigate the regulatory mechanisms underlying drug resistance-related gene expression, and ultimately suggests potential therapeutic strategies targeting lncRNA, aiming to enhance chemotherapy efficacy in tumor patients, improve patient prognosis, and provide insights for exploring novel treatment directions.

## The Role of LncRNA as a Transcriptional Regulatory Factor in Tumor Resistance

LncRNAs play an important regulatory role at multiple levels, including epigenetics, transcription, and post-transcriptional regulation, and their dysregulation is closely associated with tumor development [34]. Research has confirmed that abnormal expression of lncRNA in tumor cells influences tumor invasion, migration, and chemotherapy resistance [35]. Furthermore, studies indicate that lncRNA regulates autophagy to inhibit tumor cell apoptosis, contributing to chemotherapy resistance [36].

### LncRNA regulates tumor resistance by regulating cell autophagy

Autophagy involves transporting intracellular material to lysosomes for degradation in autophagic vesicles [37]. Autophagy, a crucial cellular process, adapts to metabolic stress, clears cellular debris (such as protein aggregates, damaged organelles, and intracellular pathogens), aids in differentiation and development, and prevents genomic damage [38,39]. In tumors, autophagy can both inhibit and support tumor progression, contributing to the development of drug resistance. Autophagy serves as a significant mechanism for drug resistance in tumors [40–42]. Regulating autophagy-related lncRNAs can restore gemcitabine sensitivity in pancreatic cancer [43]. LncRNA regulates autophagy through three different mechanisms: (1) acting as ceRNA to modulate miRNA expression, influencing autophagy; (2) altering the expression of genes associated with autophagy; (3) interacting with the Wnt/β-catenin pathway to inhibit autophagy-mediated cell apoptosis [44]. Researches [45–48] have demonstrated a strong association between gemcitabine resistance and cellular autophagy, with lncRNAs playing a critical role in its regulation. ANRIL has been shown to promote gemcitabine resistance in pancreatic cancer by downregulating miR-181a-mediated HMGB1 autophagy, thereby enhancing gemcitabine resistance [45]. LncRNA SNHG14 influences gemcitabine resistance by regulating miR-101-mediated autophagy in pancreatic cancer cells [46]. Research by Zhou et al. [47] identify that PVT1 lncRNA activates the Wnt/β-catenin pathway and autophagy, regulating miR-6195p/Pygo2 and miR-619-5p/ATG14 signaling pathways, thereby enhancing gemcitabine resistance. Tumor cells enhance chemoresistance by modulating autophagy. Autophagy is a key factor leading to gemcitabine resistance. However, the mechanism of autophagy regulation is not clear at present, which brings difficulties to clinical research on increasing tumor drug sensitivity by inhibiting autophagy. Reports indicate that the oncogenic lncRNA PVT1, acting as a ceRNA or molecular sponge, downregulates miRNA, thereby promoting tumor development and chemotherapeutic drug resistance. LncRNA PVT1 potentially regulates tumor resistance through the induction of autophagy [48]. Future research may identify lncRNA PVT1 as a novel therapeutic target for tumor resistance [49].

### LncRNA regulates tumor drug resistance by regulating tumor cell stemness

Cancer stem cells (CSCs) are a subset of tumor cells that can drive the occurrence and recurrence of tumors [50]. CSCs contribute to tumor genesis and are a fundamental cause of chemotherapy resistance. CSCs are resistant to both chemotherapy and radiotherapy, and residual CSCs after radiotherapy and chemotherapy can contribute to tumor recurrence and resistance to radiotherapy and chemotherapy [51]. Research indicates that gemcitabine treatment significantly increases the proportion of CSCs in pancreatic cancer cells [52]. LncRNAs serve as crucial regulators of CSCs and are known to enhance tumor resistance [53]. LncRNAs confer drug resistance in bladder cancer by elevating the proportion of CSCs within tumors [54]. This suggests a potential close relationship between lncRNA, CSCs, and drug resistance. LncRNA GAS5 has been shown to reverse CSC-mediated gemcitabine resistance in experiments utilizing PANC-1, ASPC-1, CAPAN-2, and SW1990 pancreatic cancer cell lines [55]. LncRNAs play an important role in drug resistance in tumor cells. Some lncRNAs can regulate tumor stem cells to reverse drug resistance [56].

### LncRNA regulates tumor drug resistance by regulating EMT

EMT refers to the important role of the cell-cell adhesion complex in embryonic development and enhances cell metastasis and invasion characteristics [57]. Tumor cells undergo this transformation to achieve metastasis and develop increased invasiveness and apoptosis resistance [58]. Studies suggest that drug-resistant cells display enhanced invasiveness, potentially linked to EMT following the development of tumor resistance [59]. LncRNAs modulate EMT transcription factors by competing with miRNAs for binding sites, influencing EMT dynamics [60]. In gallbladder cancer, lncRNAs enhance drug resistance in bile duct tumor cells through EMT regulation [61]. LncRNA regulates EMT, and it is necessary to identify the key lncRNAs that regulate EMT. Research identifies lncRNA GAS5 as a critical regulator of EMT, acting as a bait for miRNAs or splicing factors [62].

## The Role of LncRNA in Chromatin Regulation in Tumor Resistance

Cytoplasmic LncRNAs can assemble cytoplasmic complexes and isolate various cytoplasmic regulatory factors [63,64]. Intranuclear lncRNAs are attached to chromosomes by recruiting a variety of transcriptional regulatory molecules (e.g., PRC2) to the chromosome [65]. PRC2 is a trimethylation modification (H3K27me3) of three histones on LYs27, repressing the transcription of target genes [66]. However, the mechanisms by which lncRNAs regulate specific DNA sites and influence chromosome architecture remain unclear. Recent studies [67,68] suggest that lncRNAs regulate chromosome positioning via mechanisms like R-loop or triplex RNA-DNA formations.

### Protein-lncRNA

LncRNA is involved in the transcriptional regulation of several genes. LncRNA in the nucleus regulates chromatin structure, gene transcription, and RNA splicing [69]. Once in the cytoplasm, lncRNAs regulate mRNA degradation, transport, protein translation, stability, and assembly. Moreover, lncRNA is closely related to the development and prognosis of many diseases [70], such as cardiovascular and neurodegenerative diseases [71,72]. LncRNAs interact with proteins, serving roles such as protein bait, scaffold, and signaling guides, and are integral in processes including tumor development, progression, and metastasis [73]. The interaction between lncRNAs and proteins involves various mechanisms [74]: proteins with RNA binding domains (RBD) bind lncRNAs based on specific secondary structures; some proteins require sequence specificity in addition to structural features; and proteins without RBDs engage with lncRNAs through other, less understood mechanisms.

The ‘protein bait’ function of lncRNA involves regulating gene transcription, affecting chromatin stability, and altering local chromatin conformation by inhibiting protein interactions after binding. LncRNA binds to transcription factors, acting as protein bait to inhibit the transcription of genes related to signaling pathways and activation of inflammatory factors. LncRNA PANDA, mediated by p53 and DNA damage, binds to transcription factor NF-YA, blocking its recruitment to apoptosis-related genes and preventing DNA damage-induced apoptosis [75]. LncRNA also binds to chromatin-related proteins to maintain chromosome stability [76]. A typical characteristic of tumor cells is chromosomal aberration, resulting in non-diploid cells. Pumilio is a highly conserved RNA-binding protein in mammals. Pumilio1 (Pum1) and Pumilio 2 (Pum2) share high structural similarity but have overlapping targets and functions, such as embryonic development, germ cell development, cell cycle, neural response, memory, and so on [77–79], particularly in neurological degeneration [79] and acquired resistance in cancer [80,81], including ovarian cancer [82], pancreatic cancer [83], breast cancer [84], and colon cancer [85].

### LncRNA-DNA

Cell proliferation results from a series of cell cycles, the process through which a cell divides to form new cells. The cell cycle, crucial for cellular development and differentiation, comprises four phases: pre-synthesis (G1), DNA synthesis (S), post-synthesis (G2), and the division phase (M). Deregulation of the cell cycle is a primary cause of uncontrolled proliferation in cancer cells [86]. Chemotherapy drugs induce DNA damage and disrupt the cell cycle, leading to cell apoptosis. Drug resistance may develop when cells fail to recognize or repair damage, lose signaling ability for apoptosis, or have inherent defects in the apoptotic mechanisms. Key cell cycle regulatory molecules include cyclins, cyclin-dependent kinases (CDKs), and cyclin-dependent kinase inhibitors (CDKIs). Cyclins, crucial regulatory molecules, have aberrant expression patterns closely associated with cancer development [87]. CDKs are the core of cell cycle regulation, with cyclins positively regulating CDKs while CKIs negatively regulating CDKs. Together, the three constitute the molecular basis of the cell cycle regulatory network. The killing effect of tumor cells on chemotherapeutic agents is mainly achieved by direct or indirect damage to tumor cell DNA [88]. Tumor cells can better repair DNA so that it is no longer poisoned by drugs and becomes tougher. Genomic imbalance has been identified as a key factor in drug resistance. DNA damage can cause cell cycle arrest by activating cell cycle regulatory pathways and signaling pathways such as CDKs. The lncRNAs can regulate DNA damage repair functions and are involved in tumor drug resistance. HOTAIR can promote prolonged activation of NF-κB. Accordingly, lncRNAs might be involved in the resistance of ovarian cancer cells to chemotherapeutic drugs by promoting DNA damage repair [89]. Literature reports that UCA1 could regulate the sensitivity of breast cancer cells to chemotherapeutic drugs through the mTOR pathway [90]. Proteins such as MEG3 and SnaR are significantly overexpressed in colorectal cancer and other drug-resistant tumors [91]. MEG3 regulates p53 resistance to cisplatin and Bcl-XL expression [92]. Down-regulation of the above incretin RNA leads to enhanced cell proliferative activity. In lung adenocarcinoma, P21 causes DNA damage and cell cycle arrest and is a key molecule in HOTAIR-mediated cisplatin resistance. Overexpression of HOTAIR inhibits the expression of P21 and causes drug resistance [93]. HOTAIR also promotes tumor resistance in the ovary by activating signaling pathways such as NF-κ B and IL-6.

## The Role of LncRNA as a Nuclear Aggregate in Tumor Drug Resistance

The researchers recently are increasingly focusing on the interaction between lncRNA and nuclear substances [94–97]. Especially, lncRNAs regulate nucleolar activities by modulating nucleolar stress and translation reprogramming and by binding to nucleolar proteins [98–101] such as nucleolar protein (NCL) and fibrin (FBL) to control rRNA transcription and the distribution of multiribosomes. These findings highlight the vital role of lncRNAs in the transcriptional regulation of rRNA. Some lncRNAs function in both the nucleus and cytoplasm, indicating their ability to shuttle between these compartments. The presence or knockout of certain motifs leads to nuclear retention or nuclear output of lncRNA, and splicing and other RNA modifications also promote the nuclear output of lncRNA [102].

Increasing evidence suggests that nucleocytoplasmic shuttling of lncRNAs promotes tumor progression [103–105] and drug resistance [106–108]. LncRNA CASC21 binds to the transcription factor POU5F1B in the nucleus and recruits it to the promoter of growth hormone 1 (HGH1), activating the transcription of the HGH1 gene. In the cytoplasm, CASC21 also acts as ceRNA, sequestering miR-485-5P to upregulate HGH1 expression, which promotes colorectal cancer malignancy and is linked to EMT [103]. m6A modification may have an impact on the distribution of lncRNA. Overexpression of methyltransferase METTL3 increases the nuclear localization of lncRNA RP11 [104]. Interaction between RP11 and ribonucleic acid binding proteins hnRNPA2B1 accelerates mRNA degradation by two ubiquitin ligases, Siah1 and Fbxo45. Proteins with multiple ubiquitin chains are recognized and degraded by proteasomes, while ZEB1’s ubiquitination is reduced, reducing the chance of degradation by proteasomes and inducing the metastasis of colorectal cancer. When METTL3 is overexpressed, m6A induces nuclear accumulation of RP11, further promoting the upregulation of ZEB1. These studies have increased the complexity of lncRNA in tumors and laid a theoretical foundation for predicting new tumor markers, which also suggested that lncRNA may play a role in drug resistance. Moreover, there was a special lncRNA family mainly enriched in nucleolus, named small nucleolar RNA host gene (SNHG), which has important influence in tumor drug resistance. Researches [105–108] has demonstrated that SNHGs can induce chemoresistance in NSCLC cells towards drugs such as cisplatin. The resistance of tumor cells to cisplatin primarily arises from their ability to tolerate cisplatin-DNA adducts and repair DNA damage, along with factors associated with the activation of anti-apoptotic signals, active efflux of drugs from the cytoplasm, epigenetic regulation mediated by miRNAs, and immune system suppression. Ge et al. [109] identified a significant upregulation of SNHG1 in cisplatin-resistant NSCLC tissues and cells, subsequently proposing and validating its role in upregulating doublecortin-like kinase 1 (DCLK1) expression through targeting miR-330-5p, thereby augmenting the resistance of NSCLC cells to cisplatin.

## The Role of LncRNA in Post-Transcriptional Regulation in Tumor Drug Resistance

### LncRNA protein direct interaction

ATP-binding cassette (ABC) transporter proteins constitute a class of 49 membrane proteins [110]. Located in the cell membrane, they regulate drug absorption and excretion in tumor cells. Lung adenocarcinoma metastasis-related transcript 1, a highly expressed lncRNA in ovarian cancer, upregulates ABCC1 protein expression. This lncRNA, identified as LINCO1118, also negatively correlates with miR-134, known to influence paclitaxel resistance in ovarian cancer [111,112]. Therefore, lncRNAs potentially modulate chemotherapy resistance by regulating transporter protein expression levels.

### Pairing of LncRNA with other RNAs to recruit protein complexes

CeRNA is a type of RNA that shares molecular binding sites with miRNA, allowing it to bind competitively to miRNA response elements and facilitate inter-RNA communication. miRNA is an important post-transcriptional regulator. Recent studies have shown that lncRNAs can exist as ceRNAs in cells, which are closely related to chemotherapy resistance. LncRNA WDFY3-AS2 was significantly highly expressed in ovarian cancer tissues and promoted the sensitivity of ovarian cancer cells to cisplatin by competitive adsorption of miR-139-5p [113]. Transcription activating factor 3 is a carcinogenic factor, and reducing its expression increases the sensitivity of ovarian cancer cells to carboplatin chemotherapy. H19 lncRNA can function by targeting miR-29b-3p, leading to downstream target signal transduction and inhibition of transcription activating factor 3, resulting in carboplatin resistance in cancer cells [114]. Recombinant human neural calcium protein may interact with recombinant protein CX3CL1 as ceRNA, jointly promoting tumor cell infiltration and thereby promoting ovarian cancer drug resistance [115]. The miR-7 regulates LNC00115 and is closely related to miR-7. The knockdown of LNC00115 upregulated miR-7 expression in ovarian cancer and significantly inhibited cisplatin resistance in ovarian cancer. Accordingly, LNC00115 may be involved in cisplatin resistance in ovarian cancer by regulating miR-7 and modulating downstream/activated kinase subunits [116]. Multiple lncRNAs are involved in regulating ovarian cancer drug resistance by way of ceRNA. It has been reported in the literature that lncRNAs can regulate the expression of target proteins through interaction with miRNAs [89,116], which in turn affects the level of autophagy, thereby participating in tumor chemotherapy resistance. For example, lncRNA SNHG15 can target miR-381-3p to upregulate GFRAL, increasing the level of autophagy and enhancing the resistance of osteosarcoma cells to doxorubicin suggesting that autophagy can affect chemotherapy resistance [117]. LncRNA SNHG16 upregulates the expression of autophagy-related 4B genes through sponge adsorption of miR-16, thereby enhancing the relationship between autophagy and cisplatin resistance [118]. In the future, this will be a new diagnostic marker and a new therapeutic target drug resistance to improve its diagnostic and therapeutic efficacy. Guo et al. [119] found that silencing HOX in drug-resistant cell lines can down-regulate the expression of MDR genes and, through miR-106a-5p/transcriptional activator 3 expression, this affects the sensitizing effect of cisplatin on cancer cells. They explored the fact that the lncRNA HOX transcriptional antisense intergenic RNA can help improve the chemotherapy accuracy of osteosarcoma. It has been suggested that the lncRNA reprogramming factor can regulate the expression of ABC-B1 through adsorption of miR-153-3p to enhance the sensitivity of osteosarcoma to cisplatin [120]. Inhibiting lncRNA reprogramming regulatory factors may effectively avoid chemotherapy resistance. OPA interacting protein 5 antisense RNA 1 (OIP5-AS1) was able to regulate the expression of multiple growth factors by competitively binding miR-137-3p, which in turn promotes adriamycin resistance in osteosarcoma [121]. Therefore, OIP5-AS1/miR-137-3p/ growth factor axis may serve as a new pathway for reversing chemotherapy resistance in osteosarcoma [122].

### LncRNA acts as miRNA “spines”

The miRNA is a small non-coding RNA molecule, approximately 21–23 nucleotides long, with post-transcriptional regulatory functions [123,124]. Prior research has established that certain lncRNAs act as upstream regulatory factors for miRNA, playing a crucial role in tumor dynamics [125]. LncRNA, containing complementary sequences to miRNA, can competitively bind to miRNA, thus rendering the RISC silencing complex ineffective and failing to inhibit the translation of the corresponding mRNA. This mechanism, often referred to as a “miRNA sponge,” regulates tumor metastasis, recurrence, and drug resistance [126]. XIST, an additive RNA localized on the X chromosome, acts as a “sponge” for miR-204-5p, although its mechanism remains unclear [127]. Down-regulation of XIST can elevate miR-204-5p expression, enhancing cancer cell autophagy and increasing sensitivity to chemotherapeutic drugs. Wang et al.’s research [128] indicated that LINC00152 is highly expressed in hepatocellular carcinoma tissues and cells, while miR-613 expression is reduced. Functional studies demonstrated that LINC00152 can downregulate YAP1 expression via miR-613, subsequently inhibiting tumor cell proliferation, invasion, autophagy, and chemoresistance. UCA1, a lncRNA significantly upregulated in chemotherapy-resistant tumor tissues and cell lines [129], inhibits miR-513a-5p, subsequently upregulating Stathmin 1 (STMN1) expression. STMN1, a microtubule-regulating protein highly expressed in tumors [130], promotes tumor cell proliferation, invasion, and MDR; silencing UCA1 reduces STMN1 expression. PROX1-AS1, regulated by lncRNAs and associated with drug resistance, targets and regulates SOX2 expression by acting as a sponge for miR-519d-3p [131]. Following the knockdown of PROX1-AS1 or overexpression of miR-519d-3p, retinoblastoma cell proliferation, migration, and invasion were decreased.

LncRNAs can regulate miRNA expression through various mechanisms: possessing miRNA binding sites, lncRNAs can act as ceRNAs by competitively binding to specific miRNAs to form a “molecular sponge”, thereby inhibiting miRNA expression and influencing downstream target gene expression, which in turn exerts various biological functions [132,133]. This interaction is a prevalent pathway for lncRNA regulation of miRNA. In cisplatin-resistant ovarian cancer cell lines, lncRNA HOTAIR, as a ceRNA, specifically binds to miR-138-5p, thereby competing with miRNAs for the non-coding region at the 3’ end of mRNA of target genes and affecting miRNA’s regulation of target gene expression through competitive binding [133]. Some lncRNAs serve as precursors of miRNAs, generating mature miRNAs and regulating downstream gene expression to achieve specific biological functions [35,134].

## The Role of LncRNA Organelle Regulatory Function in Tumor Resistance

The endoplasmic reticulum (ER) is a multifunctional organelle crucial for various cellular processes, including protein biosynthesis and detoxification. Recent studies have highlighted that lncRNAs regulate ERS, maintaining cellular homeostasis and influencing the development of many diseases and tumors [135,136]. For instance, lncRNAs can inhibit cancer progression to malignancy by modulating the pro-apoptotic mechanisms associated with ERS. In colorectal cancer, the MEG3 lncRNA induces ERS, limits tumor cell proliferation, and enhances apoptosis, thereby preventing the malignant transformation of tumor cells [136]. These observations suggest that targeting ERS offers potential new strategies for cancer management. In particular, drug resistance in human cancers, particularly breast cancer, is becoming more closely associated with ERS [137–139]. And the ERS induced the resistance to 5-FU by the GRP78/OCT4/lncRNA MIAT/AKT pathway in breast cancer cell [140].

During disease or aging processes, mitochondrial function may deteriorate, leading to stress responses in mammals that can trigger abnormal lncRNA expression, thereby interfering with mitochondrial dynamics and cell apoptosis [141]. Maintaining mitochondrial dynamic balance is crucial for preserving the intracellular environment’s homeostasis; thus, mitochondrial dysfunction can disrupt bodily homeostasis, leading to various diseases. Mitochondrial dysfunction usually causes a shift in the energy metabolism pathway of tumour cells, making these cells more inclined to generate adenosine triphosphate (ATP) through the process of glucolysis to support their proliferation. In clinical practice, hypoxic environments also prompt tumour cells to enhance glycolysis, a phenomenon defined as the Warburg effect. This effect is often associated with increased resistance of tumour cells to drugs and tolerance to radiation therapy [142]. LncRNAs contribute to this homeostasis by regulating mitochondrial processes such as autophagy and fission and maintaining oxidative metabolism balance [143,144]. Additionally, crosstalk exists between mitochondria and lncRNAs in the nucleus and cytoplasm, which also helps maintain mitochondrial homeostasis. For example, lncRNA MALAT1 can bind to mtDNA loci in the nucleus, influencing mitochondrial function [145]. Disruption of lncRNA MALAT1 alters mitochondrial function and changes the phenotype of HepG2 liver cancer cells, while the localization of mitochondrial-encoded lncCytB is abnormal in HepG2 cells. In normal liver cells, HL7702, lncCytB is found in mitochondria, but in HepG2 cells, it is predominantly found in the nucleus [146]. This suggests that the abnormal shuttling of lncRNAs, whether encoded by the nuclear or mitochondrial genome, may lead to cellular abnormalities and affect mitochondrial metabolism. In summary, it has been discovered that mitochondrially encoded lncRNAs can maintain cellular and organelle stability by interacting with nuclear-encoded lncRNAs or by directly regulating mitochondrial functions, thereby influencing the onset and progression of tumor progress and drug resistance.

Similar as lysosomes, the main degradation organelles in eukaryotic cells, involves in processing large molecules received via endocytosis, phagocytosis, and autophagy and playing a pivotal role in promoting cellular metabolism [147,148]. By regulating these processes, lncRNAs influence the degradation or recycling of macromolecules within lysosomes. Enhanced lysosomes capacity contribute to the intracellular accumulation of exogenous drugs, such as tyrosine kinase inhibitor (TKI), thereby bolstering tumour drug resistance [149]. Moreover, lncRNAs can enhance cellular autophagy by suppressing the expression of the key autophagy regulator mTOR, thereby preventing the recruitment of mTORC1 to lysosomes [150,151]. However, research on lncRNA’s role in lysosomal membrane formation, lysosomal degradation processes, and the synthesis of lysosomal enzymes which induced tumor resistance remains limited and warrants further investigation.

## Discussion

Chemotherapy is the most common clinical treatment for malignant tumors, significantly inhibiting DNA and RNA synthesis, cell proliferation, and promoting apoptosis. However, resistance to chemotherapeutic drugs significantly impacts their effectiveness. Initial studies identified proteins associated with drug resistance, such as MDR1, ABCG2, and MRP. Recent advancements in molecular biology and high-throughput sequencing have revealed non-coding RNA profiles related to tumor drug resistance. Further investigations have shown that lncRNA interacts with and influences signaling pathways involved in tumor development and drug resistance. Identifying lncRNAs related to tumor resistance can refine treatment strategies, reducing the “experimental” use of drugs. Utilizing lncRNA inhibitors or enhancers alongside chemotherapy drugs may enhance chemosensitivity, which is particularly beneficial for patients with dosage limitations. By strategically reducing chemotherapy dosages and integrating targeted lncRNA therapy, it is possible to mitigate the adverse effects associated with high doses and reduce treatment discomfort. However, tumor resistance is multifactorial, not only due to ncRNA dysregulation but also the complexity of the tumor microenvironment. These factors contribute to discrepancies between *in vivo* and *in vitro* studies, complicating the application of lncRNA-targeted therapy to enhance chemosensitivity. Exploring the regulatory functions of lncRNAs in tumors, their mechanisms, and potential combined therapies with lncRNAs may pave the way for new treatment modalities.

## Conclusion

**Figure 1:**
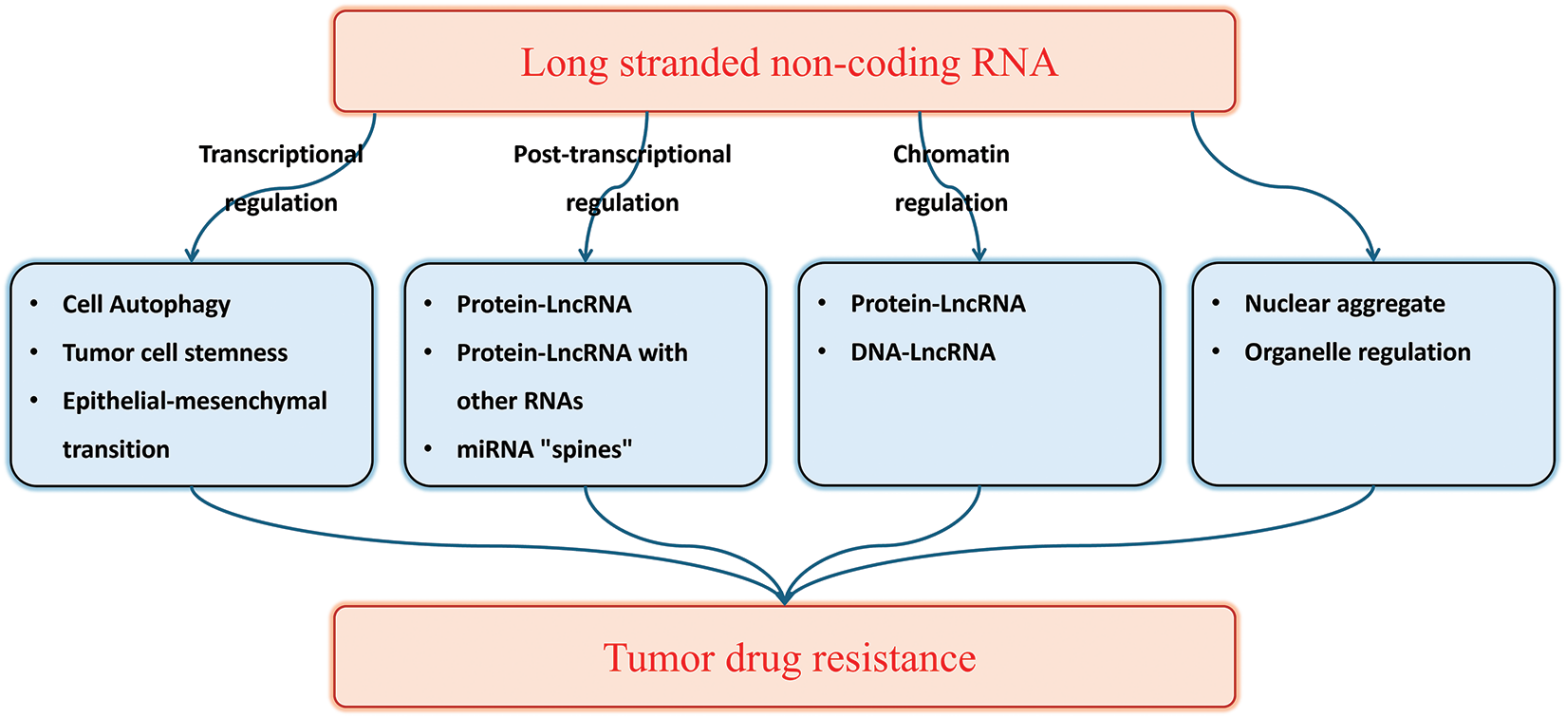
The role of lncRNA in tumor drug resistance.

Considering the extensive research on the mechanism of lncRNA in tumor drug resistance, we have made significant efforts to comprehensively summarize the involvement of lncRNA in this process. This review provides a comprehensive overview of the role of lncRNA in tumor drug resistance, as depicted in Fig. 1. Similar to refracting light through a narrow aperture, elucidating the complete panorama of this mechanism may pose challenges; nevertheless, our review establishes a robust theoretical foundation for future investigations exploring the role of lncRNA in tumor drug resistance.

## Data Availability

Not applicable.

## Abbreviations

PDR: Primary drug resistance
MDR: Multidrug resistance
GST π: Glutathione S-transferase π
ncRNA: Non-coding RNA
LncRNA: Long stranded non-coding RNA
HOTAIR: LncRNA homeobox transcript antisense intergenic RNA
EMT: Epithelial-mesenchymal transition
MALAT: Metastasis-associated lung adenocarcinoma transcript
paRNAs: Promoter-associated RNAs
eRNAs: Enhanced RNAs
ceRNAs: Competitive endogenous RNAs
Egr-1: Early growth response protein 1
miRNAs: MicroRNAs
CSCs: Cancer stem cells
CDKs: Cyclin-dependent kinase
CDKIs: Cytokinin-dependent kinase inhibitors
Pum1: Pumilio1
Pum2: Pumilio2
NBs: Nuclear bodies
pre-mRNA: Precursor mRNA
m6A: N6-methyladenosine
NCL: Nucleolar protein
FBL: Fibrin
ABC: ATP-binding cassette
OIP5-AS1: OPA interacting protein 5 antisense RNA 1
UCA1: Urothelial carcinoma associated 1
STMN1: Stathmin 1
ERS: Endoplasmic reticulum stress
